# Comparing the LC-MS Phenolic Acids Profiles of Seven Different Varieties of Brown Rice (*Oryza sativa* L.)

**DOI:** 10.3390/foods11111552

**Published:** 2022-05-25

**Authors:** Shuyi Li, Hui Xu, Yong Sui, Xin Mei, Jianbin Shi, Sha Cai, Tian Xiong, Celia Carrillo, Juan Manuel Castagnini, Zhenzhou Zhu, Francisco J. Barba

**Affiliations:** 1 School of Modern Industry for Selenium Science and Engineering, Wuhan 430023, China; lishuyisz@sina.com (S.L.); xuhui3225458@163.com (H.X.); zhenzhouzhu@126.com (Z.Z.); 2 Key Laboratory of Deep Processing of Major Grain and Oil, Ministry of Education, Wuhan 430023, China; 3 Institute for Farm Products Processing and Nuclear-Agricultural Technology, Hubei Academy of Agricultural Sciences, Wuhan 430064, China; meixin0898@163.com (X.M.); shijianbin1022@126.com (J.S.); 378079021@163.com (S.C.); xiongtian1995@126.com (T.X.); 4 National Engineering Laboratory for Deep Process of Rice and Byproducts, Central South University of Forestry and Technology, Changsha 410004, China; 5 Nutrición y Bromatología, Facultad de Ciencias, Universidad de Burgos, 09001 Burgos, Spain; 6 Preventive Medicine and Public Health, Food Science, Toxicology and Forensic Medicine Department, Faculty of Pharmacy, Universitat de València, Avda. Vicent Andrés Estellés, Burjassot, 46100 València, Spain; juan.castagnini@uv.es (J.M.C.); francisco.barba@uv.es (F.J.B.)

**Keywords:** brown rice, total polyphenol content, phenolic acids, free and bound forms

## Abstract

Brown rice, an important material of whole-grain food, is increasingly popular for its health benefits. Thus, seven varieties of brown rice from southern China were analyzed in this study, concerning the free and bound phenolic compounds in the extract. The phenolic profiles of different brown rice were obtained and compared by the combination of HPLC and LC-MS analysis, in which eleven phenolic acids were identified. It was indicated that the total phenolic contents of different brown rice varied from 92.32 to 196.54 mg of gallic acid equivalent (GAE)/100 g DW. Ferulic acid and *p*-coumaric acid, free and bound, dominated within the phenolic acids. To be mentioned, the total phenols of Luotiangongmi (a kind of red rice) were significantly higher than the other six varieties. The high phenolic content of brown rice can further guide us to explore the functional properties of the crops.

## 1. Introduction

Epidemiological studies have indicated that a healthy diet plays a vital role in preventing chronic diseases [1,2,3,4]. As we know, rice (*Oryza Sativa* L.) is the staple and main food of many developing countries. Brown rice, an important material of whole-grain food, is the product obtained from rice husking without the milling process. Compared to polished rice, the bran and embryo in brown rice remain, which contain rich nutrient substances (such as phenols, dietary fiber and minerals). It has been reported that the total sugar released from brown rice was significantly lower than that from polished rice in in vitro studies, implying that brown rice could alleviate the high glycemic responses in diabetes and hyperlipidemia patients [5]. Moreover, researchers found that the total phenolic content in bran and embryos of brown rice was 13.1 times higher than that in the endosperm [6]. It has been identified that protocatechuic acid, *p*-hydroxybenzoic acid, vanillic acid, syringic acid, *cis*-*p*-coumaric acid, ferulic acid, sinapic acid and other derivatives were present in whole grains [7,8,9], and ferulic acid was dominant in the phenolic profiles of brown rice [10,11]. Meanwhile, the conjugated phenols accounted for 40.6~50.2% of the total phenols, further proving the presence of different types of phenolic compounds in different varieties of brown rice. To be mentioned, the total phenolic content in pigmented rice was significantly higher than brown rice, in which anthocyanin/proanthocyanidin was detected [12,13]. For instance, anthocyanin-3-glucoside is the main component of anthocyanin in black rice seeds, followed by paeoniflorin-3-glucoside and anthocyanin-3-rutinoside [14]. However, Luotiangongmi, a special variety of red rice in southern China, has not been well known and studied previously.

Therefore, in this paper, seven varieties of brown rice, including Luotiangongmi from Hubei province, were selected by the Hubei Academy of Agricultural Sciences, which contained different levels of nutritional components (data not shown). Comparing the phenolic profiles of Luotiangongmi with other varieties is beneficial so to estimate its functional and economical value, and to promote the development of the local agricultural economy. The total phenolic content (TPC) involving free and bound fractions from seven varieties of brown rice were determined in this study. Furthermore, the different composition and content of the individual phenols were characterized.

## 2. Materials and Methods

### 2.1. Chemicals and Reagents

Phenolic standards such as gallic acid, protocatechuic acid, *p*-hydroxybenzoic acid, chlorogenic acid, vanillin acid, caffeic acid, syringic acid, vanillin, *p*-coumaric acid, ferulic acid and *trans*-3-hydroxycinnamic acid were purchased from Sigma-Aldrich (St. Louis, MO, USA). Acetonitrile and methanol were obtained from Fisher (HPLC-grade, Thermo Fisher Scientific, Waltham, MA, USA). Other unmarked reagents were of an analytical grade.

### 2.2. Sample Preparation

Seven varieties of brown rice (*Oryza sativa L.*): Guangliangyouxiang-66, Zhaoyou-5431, Ezhong No.5, Juliangyou-60, Y liangyou-900, Zhongzheyou No.8 and Luotiangongmi were supplied by the Rice Research Institute, Hubei Academy of Agricultural Sciences (Hubei Province, China). Guangliangyouxiang-66 and Y liangyou-900 were two-line hybrid rice; Zhaoyou-5431, Juliangyou-60 and Zhongzheyou No.8 were indica-type hybrid rice; Ezhong No.5 and Luotiangongmi were indica-type conventional rice. All varieties were freshly harvested in 2018. After dehulling, the brown rice was ground to a fine powder and sieved to a uniform particle size by passing through a 100-mesh sieve, then stored at −40 °C until analysis.

### 2.3. Extraction of Free Phenolics

The extraction of free polyphenols from brown rice was referred to a previously described method following the appropriate modifications [15]. Briefly, 2.0 g brown rice samples were mixed with 30 mL acidified methanol solution (95% methanol: 1 mol/L HCl = 90:10, *v/v*). Then, the mixture was vibrated at 50 °C by a constant-temperature shaker (Guohua, China) for 1 h, followed by centrifugation at 8000 rpm for 10 min at 4 °C. After two times of extraction, the supernatant was combined and dried by a rotary evaporator under a vacuum at 40 °C (Yarong Co., Shanghai, China). Subsequently, the dried residuals were redissolved to 10 mL with methanol (HPLC-grade) and stored at –40 °C for further analysis.

### 2.4. Extraction of Bound Phenolics

Bound phenolics were extracted according to the previous literature following appropriate modifications [16]. After removal of the free polyphenols, the remainder was collected and mixed with 20 mL 4 M NaOH for 2 h by a constant-temperature shaker. Then, the pH of the hydrolyzed product was adjusted to 2 by an HCl solution and extracted five times with ethyl acetate. The supernatant was combined and dried by a rotary evaporator under a vacuum at 40 °C. Finally, the dried residuals were redissolved to 10 mL with methanol (HPLC-grade) and stored at −40 °C for further analysis.

### 2.5. Determination of Total Phenolic Content (TPC)

The TPC of extract was determined by Folin–Ciocalteu assay with minor modifications [17]. Briefly, 0.1 mL of properly diluted extracts were mixed with 6 mL of distilled water and 0.5 mL of Folin–Ciocalteu reagent, then 1.5 mL of 10% Na_2_CO_3_ solution was added and finally diluted with distilled water to 10 mL. The mixture was incubated at room temperature for 60 min away from light, and the absorbance was measured at 765 nm using a spectrophotometer (Unic Co., Shanghai, China). The standard curve was performed with a series of gallic acid ranging from 0.1 to 0.5 mg/mL in the reaction mixture. Results of the TPC in samples were calculated and expressed as mg of gallic acid equivalents (GAE) per 100 g dry weight of brown rice (mg GAE/100 g DW).

### 2.6. UPLC-HRMS Determination of the Phenolic Composition

The monomeric phenolic acids were identified by comparing their relative retention times (RT), UV and ESI-MS spectra with authentic compounds [18,19].

The structural analysis was performed on an Ultimate 3000 ultra-high-performance liquid chromatography (UPLC) instrument coupled to a Q-Exactive Orbitrap mass spectrometer (Thermo Fisher Scientific, Waltham, MA, USA). Negative ion mode was focused by electrospray ionization (ESI) and chromatography was acquired from a Hypersil GOLD C18 column (2.1 × 100 mm, 3 µm). The column temperature was maintained at 30 °C, and mobile phase A (0.1% formic acid in acetonitrile) and B (0.1% formic acid in water) were applied. The elution procedure was as follows: 0–8 min, 5–16% A; 8–20 min, 16–35% A; 20–30 min, 35–90% A; 30–32 min, 90% A; 32–32.1 min, 90–5% A; 32.1–40 min, 5% A. The flow rate was 0.25 mL/min, and 10 µL of each sample was injected into the system. Full scans (100–1000 m/z) were acquired using the mass analyzer. In order to better respond to most of the compounds, the optimal conditions were set as follows: spray voltage, 3.2 kV; capillary temperature, 300 °C; sheath gas pressure, 40 psi; auxiliary gas pressure, 15 arb; heater temperature, 350 °C.

The main phenolic acids were simultaneously analyzed on an HPLC system according to a previous method with some modifications [20]. The chromatographic separation system was equipped with a photodiode array (PDA) detector (Waters Co., Milford, MA, USA). A C18 column (150 mm × 4.6 mm, 5 μm particle size, Shimadzu Co., Kyoto, Japan) was used and set at 30 °C. The mobile phase consisted of 0.4% acetic acid aqueous solution (A) and acetonitrile (B): 0–40 min, 5–35% B; 40–45 min, 35–50% B; 45–50 min, 50–80% B; 50–55 min, 80%–5% B; 55–60 min, 5% B. The flow rate was 1.0 mL/min, and the injection volume was 10 μL. The PDA detector scanned ranging from 200 to 400 nm. To quantify individual phenolic acid, the monitor wavelength was set at 280 nm and compared with the concentration of known standards.

### 2.7. Statistical Analysis

All experiments were repeated at least three times. The results were expressed as mean ± SD. Data were analyzed by ANOVA and *t*-test. Significance was obtained by the Duncan method. Statistical significance was declared at *p* < 0.05 or *p* < 0.01. Pearson’s correlation tests were conducted to determine the correlation between variables. Moreover, the figures were depicted with Origin 9.0 and Photoshop CC software, and the structural formulas were drawn by KingDraw 2.2 (Qingyuan Co., Qingdao, China) software.

## 3. Results and Discussion

### 3.1. Total Phenolic Content in Different Brown Rice Varieties

**Figure 1:**
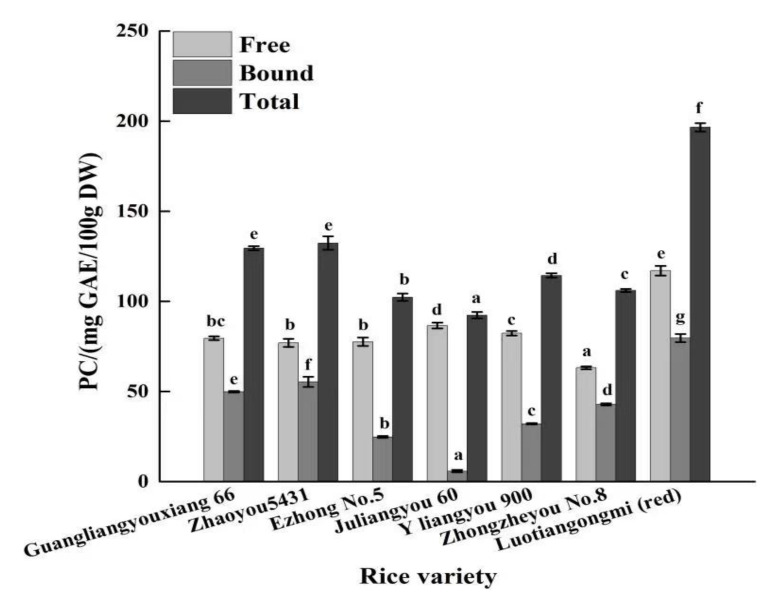
Free and bound PC in seven different varieties of brown rice. Results are expressed as mean ± SD. Percent contribution to total content is presented in parentheses. Different letters indicate significant differences among varieties for each fraction (free/bound/total) (*p* < 0.05). DW, dry weight of sample.

**Figure 2:**
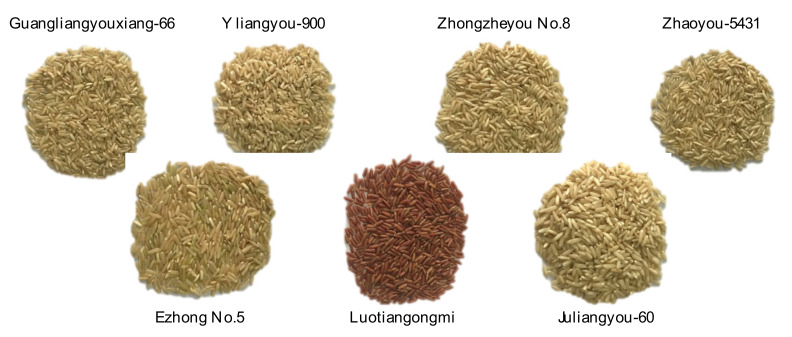
Appearance pictures of different varieties of brown rice cultivated in Hubei province.

In our understanding, the phenolic profiles of different cereal grains vary in type and variety. The free phenolic content (PC) of rice is lower than that of corn, but higher than those of wheat and oat. However, the bound PC of rice is the lowest compared to other grains [21,22]. Herein, the free and bound phenolic fractions in seven varieties of brown rice was compared, as shown in Figure 1. It was exhibited that the free PC of brown rice ranged from 63.15 (Zhongzheyou No.8) to 116.92 (Luotiangongmi) mg GAE/100 g DW, and the bound PC ranged from 5.77 (Juliangyou-60) to 79.62 (Luotiangongmi) mg GAE/100 g DW, with significant differences. It was obvious that the total PC in Luotiangongmi was significantly higher than the other varieties (*p* < 0.05), approximately 1.49~2.13 times in total quantity. The reason could be that only Luotiangongmi was the sample with a unique color in this study (Figure 2), possibly containing high amounts of anthocyanins. Moreover, the bound PC from seven varieties accounted for 6.25–41.86% of the total phenols in the extract, which is slightly lower than the result of previous literature [23]. This difference may be due to the variety of rice, the growing environment or the extraction method applied. To be mentioned, bound phenolics, also considered as insoluble phenolics, are covalently bound to cell wall structural components such as cellulose, hemicellulose, lignin and pectin, providing both physical and chemical barriers. Phenolic acids, such as hydroxybenzoic acids and hydroxycinnamic, can form ester linkages with structural carbohydrates and proteins through their carboxylic group, or generate ether linkages with lignin through their hydroxyl groups in the aromatic ring, respectively [24,25].

### 3.2. Qualitative and Quantitative Analysis of Phenolic Acids

**Figure 3:**
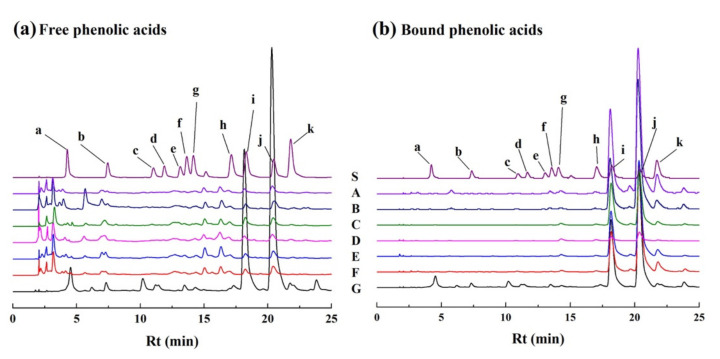
HPLC chromatograms (280 nm) of free phenolic compounds (**a**) and bound phenolic compounds (**b**) extracted from seven varieties of brown rice. (S, standard mixture of phenolic acids; A, Guangliangyouxiang-66; B, Zhaoyou-5431; C, Ezhong No.5; D, Juliangyou-60; E, Y liangyou-900; F, Zhongzheyou No.8; G, Luotiangongmi; a, Gallic acid; b, Protocatechuic acid; c, *p*-Hydroxybenzoic acid; d, Chlorogenic acid; e, Vanillic acid; f, Caffeic acid; g, Syringic acid; h, Vanillin; i, *p*-Coumaric acid; j, Ferulic acid; k, *trans*-3-Hydroxycinnamic acid).

Referring to the spectral and structural information obtained from the HPLC and LC-MS analysis, eleven phenolic acids, free and/or bound forms, were identified in different varieties of brown rice, including gallic acid, protocatechuic acid, *p*-hydroxybenzoic acid, chlorogenic acid, vanillic acid, caffeic acid, vanillin, syringic acid, *p*-coumaric acid, ferulic acid and *trans*-3-hydroxycinnamic acid (Table 1, Figure 3).

Based on the comparison to phenolic standards, the contents of these phenols and their corresponding percentage contribution in free and bound fractions were calculated and are presented in Table 2.

As reported previously, the phenolic profiles of both brown and milled rice were dominated by ferulic and *p*-coumaric acids with lower levels of gallic, vanillic, caffeic and syringic acids, which was in accordance with our study [11].

**Figure 4:**
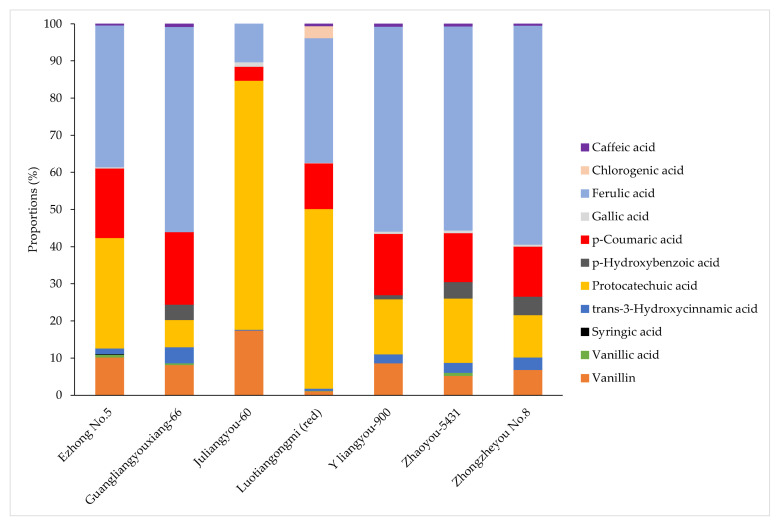
Percentual distribution of phenolics compounds extracted from seven varieties of brown rice.

In Figure 4, the distribution of phenolic compounds in each sample was presented. It can be easily observed that vanillin, *p*-coumaric acid and ferulic acid, involving free and bound forms, were present in all varieties of brown rice. However, the distribution of other phenols in the extract varied, depending on the variety and form. Gallic acid was observed in six varieties of rice except for Guangliangyouxiang-66, ranging from 1.4 (Ezhong No.5) to 4.7 (Zhaoyou 5431) μg/g DW in free forms. Free protocatechuic acid was detected in all varieties, with contents ranging from 51.3 (Guangliangyouxiang-66) to 291.0 (Luotiangongmi) μg/g DW but was only found in the bound form in the Luotiangongmi variety, with a content of 274.0 μg/g DW. Free *p*-hydroxybenzoic acid was present in Y liangyou-900 (5.4 μg/g DW), while its bound form was more abundant in Guangliangyouxiang-66, Zhaoyou 5431 and Zhongzheyou No.8, at 29.2, 28.2 and 25.0 μg/g DW, respectively. Furthermore, vanillic acid was detected in Ezhong No.5 (2.9 μg/g DW), Luotiangongmi (1.7 μg/g DW) and Zhaoyou-5431 (3.8 μg/g DW) in free forms, but its bound form was also found in Guangliangyouxiang-66 (3.4 μg/g DW). Caffeic acid existed in all varieties except Juliangyou-60 and was detected in Ezhong No.5 only in free form (1.4 μg/g DW), but in Guangliangyouxiang-66, only in bound form (5.2 μg/g DW). It is worth mentioning that some phenols were typically present in specific varieties, which can be assigned as characteristic compounds in brown rice. For example, the free and bound forms of chlorogenic acid were only found in Luotiangongmi, at 20.8 and 16.6 μg/g DW. Syringic acid only existed in the free fraction of Ezhong No.5 (0.6 μg/g DW).

In summary, Luotiangongmi (red rice) possessed the highest level of phenolic compounds, in which protocatechuic acid, ferulic acid and *p*-coumaric acid made up 50.1%, 21.3% and 24.4% of total phenols, respectively. Moreover, bound phenols play a dominant role in the phenolic compounds identified in Guangliangyouxiang-66 and Luotiangongmi. As far as vanillin was concerned, its bound fraction of Guangliangyouxiang-66 (44.3 μg/g DW) was significantly higher than other varieties (*p* < 0.05). In terms of *p*-coumaric acid, its bound form accounted for more than 90% of total phenols in all varieties, except Juliangyou-60. In the same situation, the ferulic acid presented in brown rice was mostly conjugated. According to previous research, apart from being directly connected to the cell wall, ferulic acid was also crosslinking arabinoxylan with a form of dehydrodimer as a structural component in the cell walls, playing an important role in enhancing the mechanical properties [26].

## 4. Conclusions

In order to compare the phenolic profile of different brown rice, a series of qualitative and quantitative analyses were carried out. The results indicated that the free, bound and total PC of red rice (Luotiangongmi) were significantly higher than those of brown rice (Guangliangyouxiang-66, Y liangyou-900, Zhaoyou-5431, Juliangyou-60, Zhongzheyou No.8 and Ezhong No.5). A similar trend was also found in the total FC of Luotiangongmi. Moreover, eleven kinds of phenolic compounds, including gallic acid, protocatechuic acid, *p*-hydroxybenzoic acid, chlorogenic acid, vanillin acid, caffeic acid, syringic acid, vanillin, *p*-coumaric acid, ferulic acid and *trans*-3-hydroxycinnamic acid, were determined in brown rice, in which *p*-coumaric acid and ferulic acid were comparably dominant, accounting for over 14.3% and 44.9% in total. Several phenols, such as chlorogenic acid and syringic acid, were specifically present in some varieties. In addition, the levels of bound phenolic acids were much higher than the free phenolic acids in the extracts. This study will contribute to further understanding the distribution of polyphenols in brown rice and accelerate the comprehensive utilization of red rice.

## Figures and Tables

**Table 1:** Identification of main phenolic compounds in brown rice with structural information.

Phenolic Compounds	Molecular Formula	Rt (min)	[M-H]^–^(*m/z*)	Main Fragment Ions (*m/z*)	Structure
Gallic acid	C_7_H_6_O_5_	2.22	169.0132	125.0232	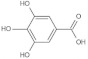
Protocatechuic acid	C_7_H_6_O_4_	3.98	153.0182	109.0283	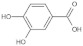
*p*-Hydroxybenzoic acid	C_7_H_6_O_3_	5.96	137.0233	93.0333	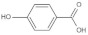
Chlorogenic acid	C_16_H_18_O_9_	6.74	353.0879	191.0555	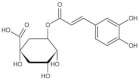
Vanillic acid	C_8_H_8_O_4_	7.26	167.0342	152.0104 123.0439 108.0204	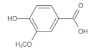
Caffeic acid	C_9_H_8_O_4_	7.52	179.0342	135.0441	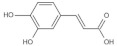
Syringic acid	C_9_H_10_O_5_	8.41	197.0447	182.0211 166.9976 152.8930	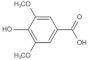
Vanillin	C_8_H_8_O_6_	9.26	151.0390	136.0155	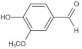
*p*-Coumaric acid	C_9_H_8_O_3_	10.20	163.0391	119.0490	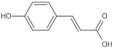
Ferulic acid	C_10_H_10_O_4_	11.28	193.0502	178.0263 149.0342	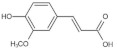
*trans*-3-Hydroxycinnamic acid	C_9_H_8_O_3_	12.21	163.0391	119.0490	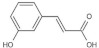

**Table 2:** Quantitative analysis of identified phenolic compounds in seven varieties of brown rice *.

Phenolic Acids	Samples	Phenolic Acid Content (μg/g DW)		
		**Free**	**Bound**	**Total**
Gallic acid	Guangliangyouxiang-66	ND	ND	ND
	Zhaoyou-5431	4.70 ± 1.60 ^b^	ND	4.70 ± 1.60 ^b^
	Ezhong No.5	1.38 ± 0.04 ^a^	ND	1.38 ± 0.04 ^a^
	Juliangyou-60	3.14 ± 1.17 ^a,b^	ND	3.14 ± 1.17 ^a,b^
	Y liangyou-900	2.83 ± 1.14 ^a,b^	ND	2.83 ± 1.14 ^a,b^
	Zhongzheyou No.8	2.77 ± 0.91 ^a^	ND	2.77 ± 0.91 ^a^
	Luotiangongmi (red)	1.62 ± 0.07 ^a^	ND	1.62 ± 0.07 ^a^
Protocatechuic acid	Guangliangyouxiang-66	51.3 ± 24.8 ^a^	ND	51.3 ± 24.80 ^a^
	Zhaoyou-5431	110 ± 4.45 ^b,c^	ND	110 ± 4.45 ^b^
	Ezhong No.5	113 ± 36.71 ^c^	ND	113 ± 36.71 ^b^
	Juliangyou-60	165 ± 21.58 ^d^	ND	165 ± 21.58 ^c^
	Y liangyou-900	68.2 ± 1.94 ^a,b^	ND	68.2 ± 1.94 ^a,b^
	Zhongzheyou No.8	57.4 ± 35.15 ^a^	ND	57.4 ± 35.15 ^a^
	Luotiangongmi (red)	291 ± 21.04 ^e^	274 ± 15.69	566 ± 28.57 ^d^
*p*-Hydroxybenzoic acid	Guangliangyouxiang-66	ND	29.2 ± 11.35 ^a^	29.2 ± 11.35 ^b^
	Zhaoyou-5431	ND	28.2 ± 8.01 ^a^	28.2 ± 8.01 ^b^
	Ezhong No.5	ND	ND	ND
	Juliangyou-60	ND	ND	ND
	Y liangyou-900	5.35 ± 0.29	ND	5.35 ± 0.29 ^a^
	Zhongzheyou No.8	ND	25.0 ± 4.73 ^a^	25.0 ± 4.73 ^b^
	Luotiangongmi (red)	ND	ND	ND
Chlorogenic acid	Guangliangyouxiang-66	ND	ND	ND
	Zhaoyou-5431	ND	ND	ND
	Ezhong No.5	ND	ND	ND
	Juliangyou-60	ND	ND	ND
	Y liangyou-900	ND	ND	ND
	Zhongzheyou No.8	ND	ND	ND
	Luotiangongmi (red)	20.8 ± 2.86	16.6 ± 3.34	37.3 ± 6.16
Vanillic acid	Guangliangyouxiang-66	ND	3.37 ± 2.33 ^a^	3.37 ± 2.33 ^a,b^
	Zhaoyou-5431	3.75 ± 1.02 ^b^	1.64 ± 0.17 ^a^	5.39 ± 1.17 ^b^
	Ezhong No.5	2.87 ± 0.30 ^a,b^	ND	2.87 ± 0.30 ^a,b^
	Juliangyou-60	ND	ND	ND
	Y liangyou-900	ND	ND	ND
	Zhongzheyou No.8	ND	ND	ND
	Luotiangongmi (red)	1.65 ± 0.24 ^a^	ND	1.65 ± 0.24 ^a^
Caffeic acid	Guangliangyouxiang-66	ND	5.16 ± 1.45 ^c^	5.16 ± 1.45 ^d^
	Zhaoyou-5431	0.70 ± 0.50 ^a^	3.24 ± 0.84 ^b^	3.95 ± 0.75 ^c,d^
	Ezhong No.5	1.37 ± 0.69 ^a,b^	ND	1.37 ± 0.69 ^a^
	Juliangyou-60	ND	ND	ND
	Y liangyou-900	1.67 ± 0.26 ^b^	1.54 ± 1.12 ^a^	3.21 ± 0.88 ^b,c^
	Zhongzheyou No.8	0.99 ± 0.30 ^a,b^	1.20 ± 0.34 ^a^	2.19 ± 0.09 ^a,b^
	Luotiangongmi (red)	0.78 ± 0.16 ^a^	5.91 ± 0.30 ^c^	6.69 ± 0.42 ^e^
Syringic acid	Guangliangyouxiang-66	ND	ND	ND
	Zhaoyou-5431	ND	ND	ND
	Ezhong No.5	0.56 ± 0.11	ND	0.56 ± 0.11
	Juliangyou-60	ND	ND	ND
	Y liangyou-900	ND	ND	ND
	Zhongzheyou No.8	ND	ND	ND
	Luotiangongmi (red)	ND	ND	ND
Vanillin	Guangliangyouxiang-66	13.2 ± 3.21 ^a,b^	44.3 ± 16.22 ^b^	57.5 ± 19.43 ^c^
	Zhaoyou-5431	9.01 ± 3.79 ^a^	24.6 ± 9.94 ^a^	33.6 ± 6.80 ^a,b^
	Ezhong No.5	18.3 ± 6.88 ^a,b^	20.6 ± 5.39 ^a^	38.9 ± 12.27 ^b,c^
	Juliangyou-60	21.7 ± 8.21 ^b^	21.2 ± 5.02 ^a^	42.9 ± 13.00 ^b,c^
	Y liangyou-900	18.1 ± 4.04 ^a,b^	22.1 ± 12.15 ^a^	40.2 ± 16.16 ^b,c^
	Zhongzheyou No.8	13.4 ± 7.83 ^a,b^	21.2 ± 5.02 ^a^	34.6 ± 3.34 ^a,b,c^
	Luotiangongmi (red)	13.4 ± 1.14 ^a,b^	ND	13.4 ± 1.14 ^a^
*p*-Coumaric acid	Guangliangyouxiang-66	1.49 ± 0.02 ^a^	135 ± 2.46 ^e^	137 ± 2.46 ^e^
	Zhaoyou-5431	1.57 ± 0.34 ^a^	82.4 ± 1.95 ^d^	83.9 ± 2.13 ^d^
	Ezhong No.5	3.73 ± 0.94 ^b^	67.3 ± 0.40 ^b^	71.1 ± 1.33 ^b^
	Juliangyou-60	2.40 ± 0.72 ^a^	6.72 ± 0.71 ^a^	9.12 ± 1.43 ^a^
	Y liangyou-900	2.11 ± 0.64 ^a^	73.9 ± 2.98 ^c^	76.0 ± 3.05 ^c^
	Zhongzheyou No.8	2.04 ± 0.64 ^a^	65.8 ± 3.68 ^b^	67.8 ± 3.55 ^b^
	Luotiangongmi (red)	1.49 ± 0.46 ^a^	142 ± 3.97 ^f^	144 ± 3.92 ^f^
Ferulic acid	Guangliangyouxiang-66	4.32 ± 0.73 ^b,c^	384 ± 11.76 ^f^	388 ± 11.08 ^f^
	Zhaoyou-5431	5.71 ± 0.24 ^d^	345 ± 12.11 ^e^	350 ± 12.21 ^e^
	Ezhong No.5	5.53 ± 0.87 ^c,d^	139 ± 0.78 ^b^	145 ± 0.20 ^b^
	Juliangyou-60	4.55 ± 1.02 ^b,c,d^	20.6 ± 0.23 ^a^	25.1 ± 0.79 ^a^
	Y liangyou-900	4.21 ± 0.26 ^b^	251 ± 11.82 ^c^	255 ± 11.83 ^c^
	Zhongzheyou No.8	5.32 ± 0.17 ^b,c,d^	291 ± 18.11 ^d^	296 ± 11.28 ^d^
	Luotiangongmi (red)	2.47 ± 0.78 ^a^	392 ± 9.21 ^f^	394 ± 9.87 f
*trans*-3-Hydroxycinnamic acid	Guangliangyouxiang-66	0.18 ± 0.07 ^a,b^	30.3 ± 1.89 ^d^	30.5 ± 1.89 ^e^
	Zhaoyou-5431	0.44 ± 0.02 ^d^	16.9 ± 1.63 ^c^	17.3 ± 1.62 ^d^
	Ezhong No.5	0.33 ± 0.08 ^c,d^	5.61 ± 1.36 ^a^	5.94 ± 1.36 ^b^
	Juliangyou-60	0.58 ± 0.15 ^e^	ND	0.58 ± 0.15 ^a^
	Y liangyou-900	0.29 ± 0.01 ^b,c^	11.0 ± 2.01 ^b^	11.2 ± 2.01 ^c^
	Zhongzheyou No.8	0.08 ± 0.01 ^a^	16.7 ± 1.02 ^c^	16.8 ± 1.02 ^d^
	Luotiangongmi (red)	0.40 ± 0.03 ^c,d^	6.75 ± 3.38 ^a^	7.14 ± 3.38 ^b^

* Results are expressed as mean ± SD. Different letters indicate significant differences among varieties for each phenolic acid and fraction (free/bound/total) (*p* < 0.05). DW, dry weight of sample. ND, not detected.

## Data Availability

The data presented in this study are available in article.
